# Do drowning and anoxia kill head lice?

**DOI:** 10.1051/parasite/2018015

**Published:** 2018-03-09

**Authors:** Kerdalidec Candy, Sophie Brun, Patrick Nicolas, Rémy Durand, Remi N. Charrel, Arezki Izri

**Affiliations:** 1 Parasitology-Mycology Department, Avicenne Hospital, AP-HP, Bobigny France; 2 Unit of Pharmacology, Avicenne Hospital, AP-HP, Bobigny France; 3 Unité des Virus Emergents (Aix-Marseille Univ – IRD 190 – Inserm 1207 – IHU Méditerranée infection), Marseille France

**Keywords:** *Pediculus humanus capitis*, drowning, water, anoxia, oxygen

## Abstract

Chemical, physical, and mechanical methods are used to control human lice. Attempts have been made to eradicate head lice *Pediculus humanus capitis* by hot air, soaking in various fluids or asphyxiation using occlusive treatments. In this study, we assessed the maximum time that head lice can survive anoxia (oxygen deprivation) and their ability to survive prolonged water immersion. We also observed the ingress of fluids across louse tracheae and spiracle characteristics contrasting with those described in the literature. We showed that 100% of lice can withstand 8 h of anoxia and 12.2% survived 14 h of anoxia; survival was 48.9% in the untreated control group at 14 h. However, all lice had died following 16 h of anoxia. In contrast, the survival rate of water-immersed lice was significantly higher when compared with non-immersed lice after 6 h (100% vs. 76.6%, *p* = 0.0037), and 24 h (50.9% vs. 15.9%, *p* = 0.0003). Although water-immersed lice did not close their spiracles, water did not penetrate into the respiratory system. In contrast, immersion in colored dimeticone/cyclomethicone or colored ethanol resulted in penetration through the spiracles and spreading to the entire respiratory system within 30 min, leading to death in 100% of the lice.

## Abstract (fr)

Des méthodes chimiques, physiques et mécaniques sont utilisées pour contrôler les poux humains. Des tentatives ont été faites pour éradiquer les poux de tête *Pediculus humanus capitis* par l’air chaud, en les trempant dans divers fluides ou en les asphyxiant à l’aide de traitements occlusifs. Dans cette étude, nous avons évalué le temps maximum que les poux de tête peuvent survivre à l’anoxie (privation d’oxygène) et leur capacité à survivre à une immersion prolongée dans l’eau. Nous avons également observé la pénétration de fluides à travers les trachées des poux et les caractéristiques du spiracle, contrastant avec celles décrites dans la littérature. Nous avons montré que 100 % des poux supportent 8 h d’anoxie et que 12,2 % survivent à 14 h d’anoxie ; 48,9 % de survie à 14h a été obtenu dans le groupe témoin non traité. Cependant, tous les poux sont morts après 16 h d’anoxie. En revanche, le taux de survie des poux immergés était significativement plus élevé que celui des poux non immergés après 6 h (100 % contre 76,6 %, p = 0,0037) et 24 h (50,9 % contre 15,9 %, p = 0,0003). Bien que les poux immergés dans l’eau n’aient pas fermé leurs spiracles, l’eau n’a pas pénétré dans le système respiratoire. En revanche, l’immersion dans de la diméticone/cyclométhicone colorée ou de l’éthanol coloré a entraîné une pénétration à travers les spiracles et une propagation à l’ensemble du système respiratoire dans les 30 minutes, entraînant la mort de 100 % des poux.

## Introduction

Lice are obligate human hematophagous ectoparasites belonging to the Pediculidae family [14,54]. Pediculosis infestation due to head lice (*Pediculus humanus capitis* De Geer, 1778) has a global distribution and affects individuals of broad economic or social status worldwide, particularly school-aged children [19,25]. Several studies have reported the presence of *Acinetobacter* spp., *Bartonella quintana*, and *Borrelia recurrentis* in head lice [2,5–6]. Furthermore, laboratory experiments have demonstrated the ability of head lice to transmit pathogens such as *Rickettsia prowazekii* [23,41,49] but whether or not there is a medical impact is still debated. Pediculosis capitis is also a source of social concern worldwide. Head lice may induce skin irritation, superinfection from scratching, social stigmatization, and psychological distress [17,24,39]. The economic implications are also substantial [26,27,40].

Different chemical, physical, and mechanical methods are used to control pediculosis. Use of insecticides such as pyrethrin or malathion was the treatment of choice until recently [7,16]. However, alternative treatments [8,9,28] are currently replacing insecticide use due to widespread resistance to neurotoxic agents [18]. Since the early 2000s, different physical acting treatments based on various oils, alcohol or plant extracts have been tested *in vitro* or in clinical trials [1,7,10,21,34,35,43,44,48,57]. Some products appear to asphyxiate nits and lice by blocking air uptake at the surface [35,44], whereas others achieve this by penetrating the tracheal system [1,48]. However, whether or not death was caused by asphyxiation remains under debate [10,11].

The efficacy of lice infestation treatment has been compared using a variety of protocols, including: hot and cold air [13,22], machine laundering [30,36,51], immersion in water [4,38,52], immersion in chemical agents [3], and immersion in ethanol [37,50]. The efficacy of chemical agents and ethanol has been demonstrated [3,37,50], whereas the efficacy of water remains controversial. Moreover, controversy exists concerning the role of spiracles during immersion.

Accordingly, we conducted the following experiments to investigate: (i) how long head lice can survive under anoxic conditions, (ii) the ability of head lice to survive up to 24 h when immersed in water, (iii) the closure of their spiracles when immersed in water, and (iv) the penetration of water and other products into the respiratory system of head lice.

## Materials and Methods

### Ethics

The protocol was reviewed and approved by the *Comité de Protection des Personnes* (institutional review board) of the Ethics Committee CPP- Ile-de-France X (2017-02). Informed consent was obtained from all patients.

### Collected lice

From September 2015 to July 2016, *Pediculus humanus capitis* were obtained by combing the hair of infested patients of 2–68 years of age attending the parasitology department of the Avicenne Hospital, Bobigny, France. Head lice were placed in plastic Petri dishes and were examined for activity and morphological integrity under a binocular magnifier (Zeiss Stemi 2000C). Tests were performed using living, non-injured lice. We started experiments immediately after lice collection. All experiments were done under the same conditions of diet, temperature of water (25 ± 2 °C), and ambient air (25 ± 2 °C, 20–50% relative humidity).

### Anoxia bioassay

To evaluate the effect of anoxia on head lice survival, ten groups of 30 lice each were used. Each group (n = 30) was placed in a plastic Petri dish (5 cm in diameter) and was placed in an AnaeroGen^TM^ Compact plastic pouch (Oxoid Ltd, Basingstoke, England, UK). Then, the AnaeroGen^TM^ Compact sachet (Oxoid Ltd, Basingstoke, England, UK) was quickly placed in the pouch. The system was tied shut with a clip. Each group endured anoxia for one time point (H1, H3, H6, H8, H10, H12, H14, H16, H18, and H24). Each test group had a control group (n = 30) that was placed in a plastic Petri dish under ambient conditions (25 ± 2 °C, 20-50% relative humidity). The pouches were then opened and viable and dead lice were counted separately (≤ 1h) for the test and control groups, respectively. Death was defined as the absence of vital signs such as movement of antennae, movements of legs, and gut peristalsis. Each experiment was performed in triplicate.

### Lice survival after water immersion

Seven groups of lice (n = 7–18 each) were placed in plastic Petri dishes (5 cm in diameter) and kept immersed in 10 mL of tap water (24 °C) for H1 to H6, and H24. Petri dishes were gently agitated until all lice were completely submerged and resting at the bottom, and then covered with their lid to prevent water evaporation. After the immersion period, water was removed and live or dead lice were counted. Seven groups of control lice (n = 7–18 each) were placed in Petri dishes without water under room conditions (25 ± 2 °C, 20-50% relative humidity) for the corresponding duration (H1 to H6, and H24) and were processed similarly. The 7 to 18 lice used in each group were of both sexes. The experiments were performed in triplicate. Death was defined as described above.

### Effect of water immersion on lice spiracles

Three groups of lice (n = 3 each) were placed into a 1.5 mL microfuge tube containing 1 mL of tap water and kept immersed for 10 min, 3 h or 24 h. Control groups were placed into 1.5 mL tubes without water. Upon completion, lice were frozen at −20 °C to preserve the status of the spiracles (SEM1). A similar series of experiments (SEM2) was performed except that −20 °C refrigeration was replaced by liquid nitrogen immersion to avoid rapid movement of the spiracles. After a rapid thaw, specimens were mounted on aluminium stubs on double-sided conductive carbon tabs and positioned on the stubs using a stereoscopic microscope. Samples were analyzed using a FEI Quanta FEG 250 environmental scanning electron microscope operating in LowVac mode, enabling the observation of resin coated samples without metallization and ESEM mode, which is normally used for the observation of wet samples (Technology University of Compiègne, France). The chamber pressure was 3 Torr, the Horizontal Field Width was 63.5 μm, and the working distance was 10 mm with a tilt of −1 °. The images were recorded at 1536 x 1103 pixel magnification.

### Penetration of water, dimeticone/cyclomethicone and ethanol into the respiratory system

Penetration or absence of penetration of liquids through the spiracles into the respiratory system was studied using a water-based stain, an absolute ethanol-based stain, and dimeticone/cyclomethicone-based stain (Pouxit^®^, Cooper, France). The latter contains 4% w/w dimeticone in a volatile silicone base (cyclomethicone 5).

The stain (0.1%) in water and absolute ethanol was brilliant blue FCF (E133) (Meilleurduchef, France). The stain (1%) in dimeticone/cyclomethicone was a red lipophilic colorant (FluoTecknic, France).

Nine groups of lice (n = 3 each) were placed into a 1.5 mL microfuge tube containing either (i) 1 mL of blue tap water or 1 mL of blue absolute ethanol, or (ii) 1 mL of red dimeticone/cyclomethicone. Lice were kept immersed for 10 min, 30 min or 12 h. Controls consisted of 9 groups of lice (n = 3 each), which were processed identically, except for the absence of dye. After immersion, lice were dried gently on a filter paper to absorb excess of fluid and then abundantly rinsed with tap water before examination under a binocular magnifier.

### Statistical analysis

The survival of lice after immersion or anoxia bioassays was expressed as the percentage of living specimens. For immersion and anoxia bioassays, the Fisher’s exact test was used to compare the survival rate between groups, looking for a relationship with time of immersion or exposure by the Cochran-Mantel-Haenszel tests. For anoxia bioassay, the 3-way ANOVA model with interaction was used to check whether the treatment (anoxia or control) remains independent of the time of exposure. The analyses were carried out at the two-tailed *p* < 0.05 level using JMP version 10.0.0 (SAS Institute, Cary, NC, USA).

## Results

### Anoxia bioassay

**Figure 1:**
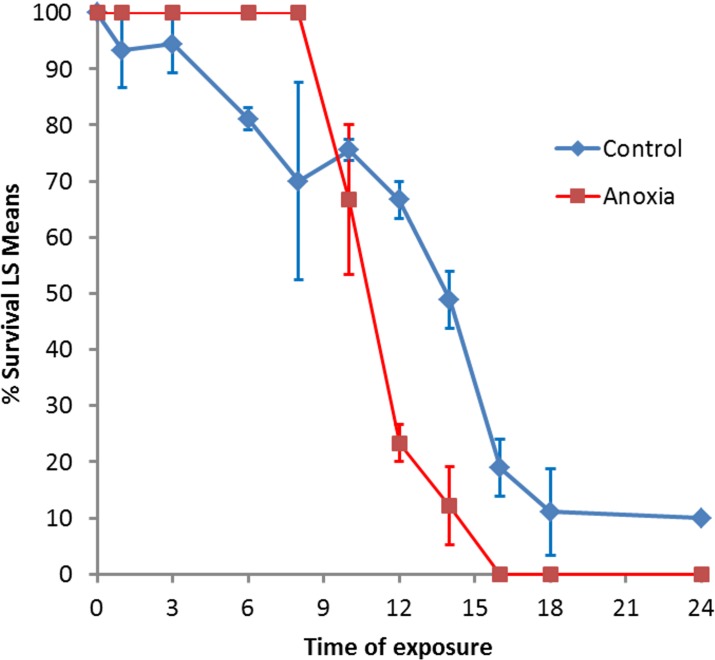
Survival of head lice after anoxia exposure compared with control lice maintained at ambient temperature.

A total of 1,787 head lice were used for this study. In all, 900 lice were held under anoxic conditions and 887 lice were used as untreated controls. Those placed in the anaerobic pouches remained mobile for at least 5 min. After 10 min, intestinal peristalsis and legs/antennae movement showed decreased activity. After 30–40 min, all lice appeared completely immobile. After extraction from the anaerobic pouches at H1, H3, H6 or H8, 100% (360/360) of lice were alive compared with 87.1% (305/350) in the matched control groups (*p* < 0.0001). After H10, death was observed in the anoxic group (Figure 1). At H14, the survival rate in the anoxic group was lower 12.2% (11/90) than in the control group 48.9% (44/90). All treated lice were dead after H16 vs survival rates of 18.9% (17/90) at H16, 11.1% (10/90) at H18, and 10% (9/90) at H24 in the untreated control groups.

### Survival of lice following immersion in water

**Figure 2:**
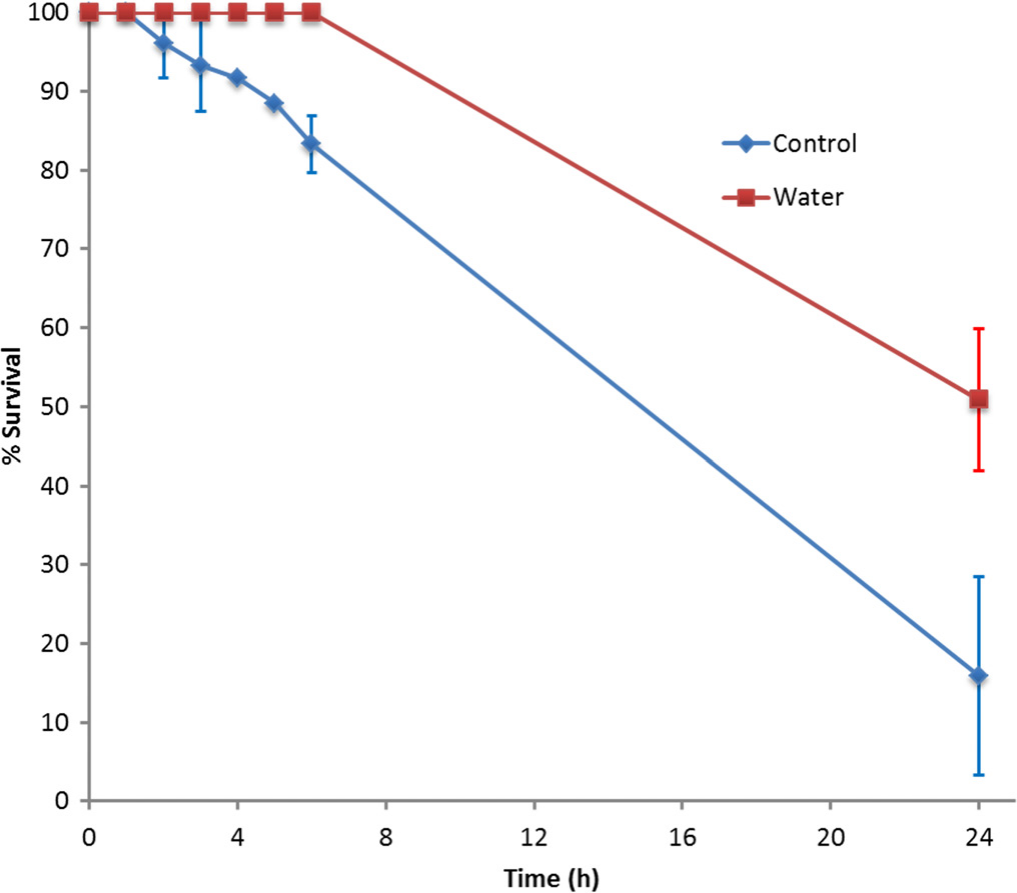
Survival of head lice after immersion in water for 1 h to 6 h, and 24 h compared with control lice maintained at ambient temperature.

A total of 503 head lice were used, of which 243 were immersed in water and 260 were used as untreated controls. Following immersion in water, antennae and leg movement of all lice ceased within less than 1 min, while intestinal peristalsis persisted for up to 10 min, then lice appeared completely immobile. However, none of the 188 lice immersed between H1 and H6 were dead, despite their apparent immobility. After H24, 50.9% (28/55) of water-immersed lice were alive. Mortality was higher in the control group compared with the test group (Figure 2). The dynamics of recovery were recorded; gut peristalsis and movements of antennae resumed within 5 min following 1-3 h immersion, and after 10 min following 4-6 h immersion. For those immersed for 24 h, 23.6% (13/55) showed slight movement of legs, antennae or gut peristalsis when released from the immersion conditions; 20 min later, the whole body convulsed [43.6% (24/55)] and after 1 hour, 50.9% (28/55) of these lice were alive and fully active.

### Effect of water immersion on lice spiracles

**Figure 3:**
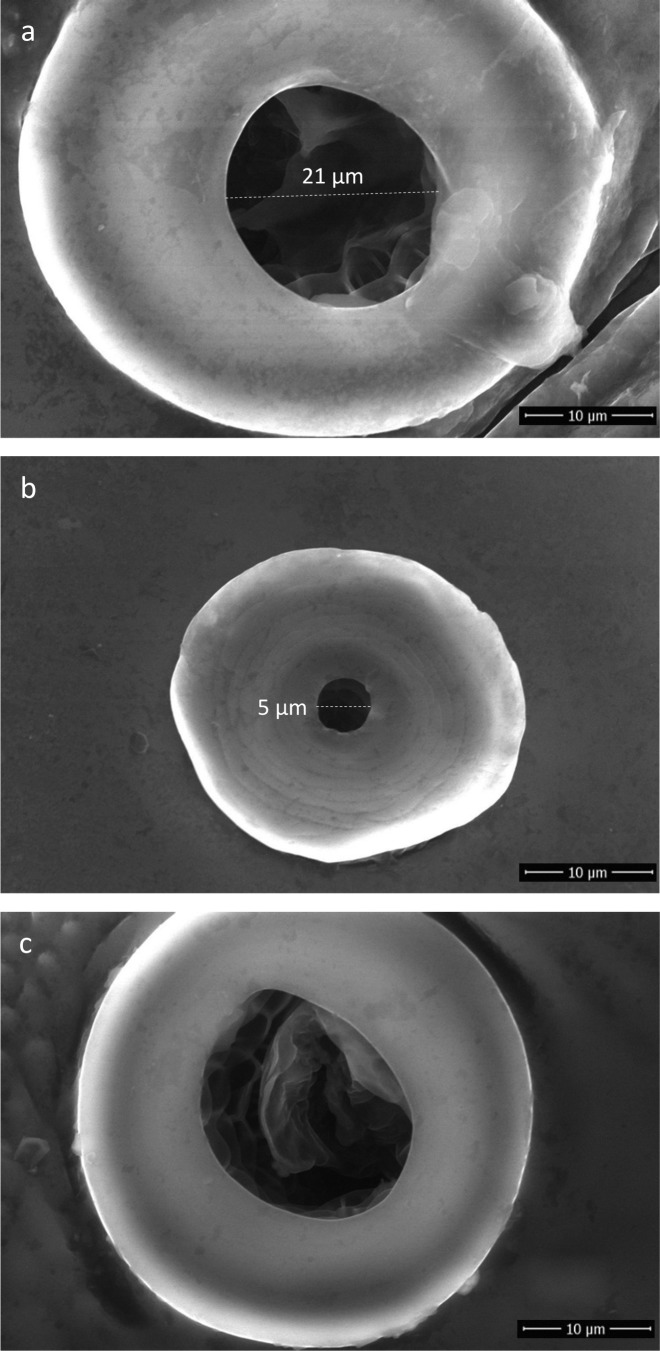
Representative scanning electron micrographs showing morphology of head lice spiracles after 24 h water immersion: a) Thoracic spiracle, b) Abdominal spiracle, c) Thoracic spiracle showing secreted material.

A total of 36 lice were aliquoted into 6 study groups (n = 3 each) and 6 untreated control groups (n = 3 each). Irrespective of the specific experiment (water-immersed or non-immersed) and the freezing method (−20 °C or −196 °C), the diameter of the thoracic and abdominal spiracles measured approximately 20 μm (Figure 3a) and ∼5 μm (Figure 3b), respectively. We also noted that abdominal and thoracic spiracles from water-immersed lice contained a secreted material (Figure 3c).

### Penetration of water, dimeticone/cyclomethicone or ethanol into the respiratory system

**Figure 4:**
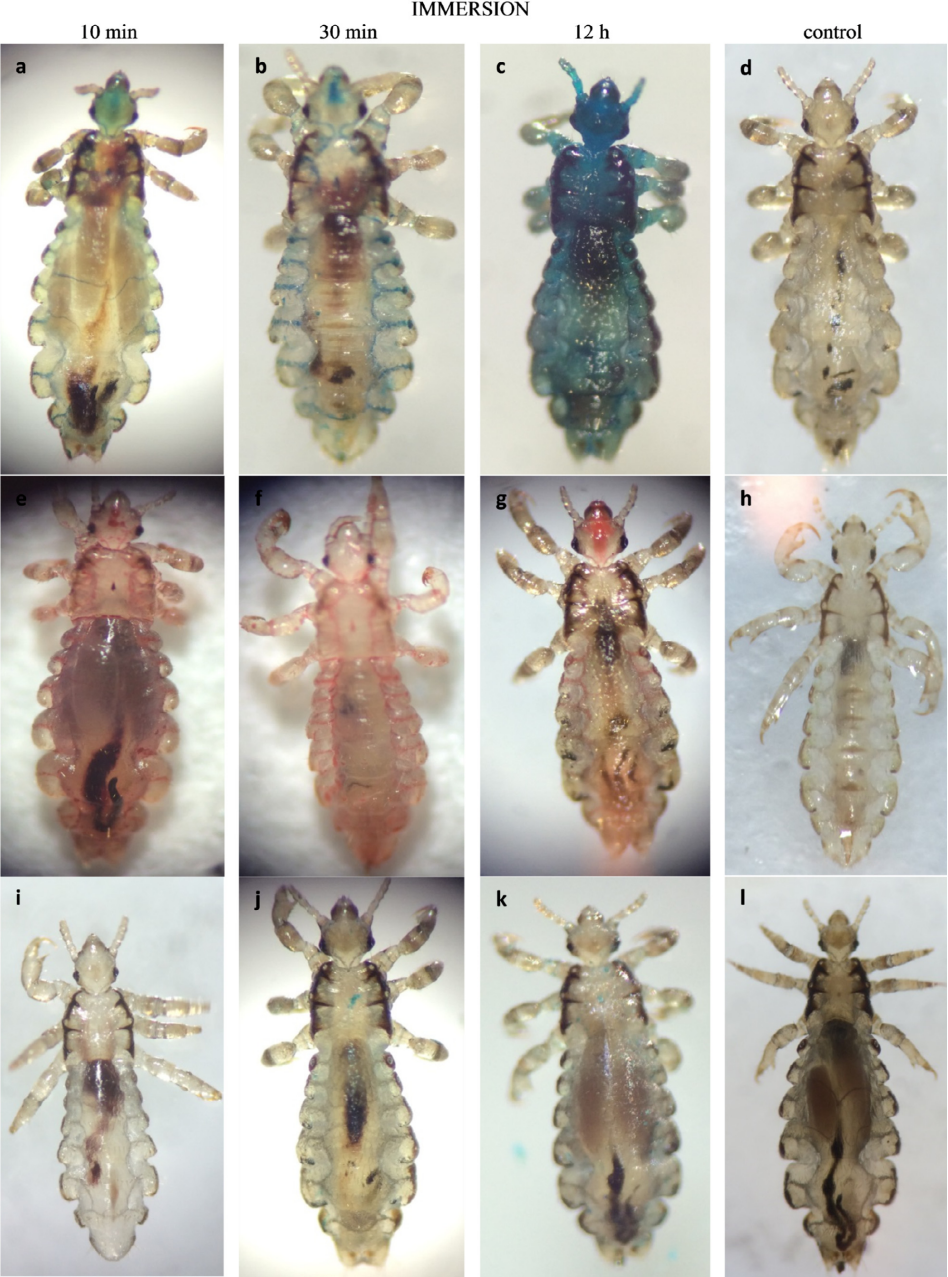
Photographs of head lice immersed in ethanol (first row), in dimeticone/cyclomethicone (second row) or in water (third row). Dark-blue stained ethanol entered across spiracles and filled the respiratory system (a, b, c), control (d). Red-stained dimeticone/cyclomethicone entered across spiracles and filled the respiratory system (e, f, g), control (h). Dark blue stained water did not penetrate the respiratory system (i, j, k), control (l).

A total of 54 head lice were used for this study. All lice immersed in water containing stain (Figure 4i, j, k) showed a total lack of coloration of the tracheal system regardless of the duration of contact (10 min, 30 min, or 12 h). On the other hand, colored ethanol stained the lice spiracles within 10 min of contact (Figure 4a) and the entire tracheal system was colored after either 30 min or 12 h (Figure 4b, c). Likewise, colored-dimeticone/cyclomethicone stained the lice spiracles within 10 min of contact (Figure 4e). After 30 min and 12 h, the entire tracheal system of the head lice was red-colored, indicating that the stained-dimeticone/cyclomethicone had filled the tracheal system (Figure 4f, g). Lice immersed in colored or non-colored dimeticone/cyclomethicone or 100% ethanol were all dead following immersion for 30 min.

## Discussion

### General observations

Head lice cause skin irritation, superinfection from scratching, and might also transmit severe infectious diseases under endemic or epidemic conditions [2]. For centuries, humans have attempted to combat head lice infestation. Remnants of these practices can be observed in non-human primates in which de-lousing has turned into a social activity. In response to increasing resistance encountered with neurotoxic insecticides, alternative treatments relying on physical properties, rather than chemicals, have been developed and evaluated either *in vitro*, clinically or both. Most of these treatments are based on oils, alcohol or plant extracts [1,7,10,21,34,35,43,44,48,57]. The claimed mechanisms of action of these compounds rely either (i) on blocking the penetration of the compounds at the surface of the nits and lice [35,44], or (ii) on penetration of the trachea [1,48]. Since these compounds block air uptake by lice, it was extrapolated that death occurred by asphyxiation/suffocation. However, the precise mechanisms leading to death in these conditions has been questioned [10,11]. For these reasons, we decided to investigate whether or not anoxia was effective against lice and for how long lice could endure oxygen deprivation. We also evaluated lice survival after immersion in water, to study whether or not their respiratory system was protected by closing the spiracles, and how effective the spiracles were at preventing entry of liquid into the respiratory system.

### Anoxia bioassay

Data to quantify how long head lice can survive under anoxic conditions are lacking. This study showed that oxygen deprivation caused higher mortality rates of all groups subjected to this condition for >8 h, when compared with untreated controls (survival 12.2% *vs* 48.9%, *p* < 0.0001). The fact that after 14 h of anoxia, only 12.2% of lice were alive compared with 48.9% of controls demonstrates that either long-term oxygen deprivation or carbon dioxide increases, ranging from 8 to 16% (after 24 h /or both) kill lice. The AnaeroGen^TM^ sachet reduces oxygen to < 0.1% within 2.5 h, while generating 8–16% carbon dioxide, suggesting that lice are adapted to total oxygen deprivation for 10-12 h. Death of lice by asphyxiation was claimed for several physically active treatments [1,35,44,48]. Depending on the concentrations used and the exposure period, carbon dioxide may have a variety of impacts on insects including bait, anesthesia, and pest control. Additionally, the carbon dioxide effect may vary from being innocuous to having a severe impact on lice survival depending on concentration, temperature, insect species, or other factors [29,45,46]. In contrast to reported studies that used either very high carbon dioxide concentrations for a short period of time or low concentrations for longer exposure periods, we maintained lice for < 24 h in the AnaeroGen^TM^ pouch where carbon dioxide levels never exceeded 16%. After 16 h of anoxia, the death rate was very high. A limitation of our study was that controls were maintained in an ambient atmosphere rather than in anaerobic pouches, i.e. without use of the AnaeroGen^TM^ sachet. Thus, the high mortality rate in the untreated controls during the first few hours could be due to dehydration.

### Lice survival after water immersion

When immersed in water, we observed a survival rate of 100% after 6 h, and 50% after 24 h; during these experiments louse mobility ceased totally. The latter observation had also been reported in previous studies with head lice [15] and amphibious lice (*Antarctophthirus microchir*) [32]. Our results are of the same magnitude as described for body lice by Mumcuoglu *et al*. [38] (100 % mortality rate after 19 h in 24 °C water). At −1 °C, body lice can survive for up to 67 h [4]. For head lice, there was only one previous study that reported a much higher mortality after water immersion (87% and a 100% death rate after 12 h and 16 h, respectively) [53]. Takano-Lee *et al*. [53] observed that 13% (n = 8) of head lice recovered following water immersion for 12 h, but that 100% (n = 20) of lice were killed after 16 h of immersion. The fact that mortality was higher in untreated controls than in immersed lice may reflect the total immobility induced by water immersion; since water is not rapidly toxic for lice, the absence of movement reduces metabolic activity, which could otherwise be deleterious if combined with starving; in addition, control lice may also have suffered from dehydration. Whether head lice, immersed in water, die from anoxia or from drowning due to penetration of water into their respiratory system was never investigated.

### Effect of water immersion on louse spiracles

From the outset of this study, it was common knowledge that head lice immersed in water protect their respiratory system from water penetration either by closing their spiracles [3,12,31,33,34], or by closing hypothetical anatomic structures located below the spiracles [14,20,42,55]. However, these two putative mechanisms have not been further documented and remain hypothetical. Meinking *et al*. [34] observed that lice immersed in mayonnaise sauce presented clogged spiracles but no evidence of closed spiracles. Buxton [14], Ferris [20], Nuttall *et al*. [42], and Webb [55] described an occlusor muscle in Anoplura species, including *Pediculus*. It is generally accepted that the inner occlusor muscle in Anoplura species works to close the spiracles [14,20,42,55], in order to restrict water loss [14]. However, to date, the exact function of this muscle remains obscure. In our study, we observed (i) that the trachea was not closed and presented a honeycomb internal structure, and (ii) the presence of a secreted material in spiracles as well as glands at the base of the spiracles that might be responsible for the production of this substance. However, detailed studies are needed to characterize both glands and material in more detail.

Our results showed that blocking water penetration into the respiratory system of head lice is not mediated by physically closing the spiracles or decreasing their diameter. Freezing at −20 °C is a relatively slow process and might result in erroneous conclusions concerning the condition of spiracles at any precise moment. For this reason, and to avoid any bias due to instant contraction of the spiracles, if any, a second experiment was performed using liquid nitrogen (−196 °C) that results in instant freezing of the physical state of spiracles during immersion. Interestingly, modification of the diameter of spiracles through slow or instant closing was not observed in our study. There was no difference in the size of spiracle diameter when lice were in the air or in water; prevention of water penetration probably relies on an alternative mechanism. This contradicts previous reports, which on examination brought little insight on the mechanism itself [3,12,31,33].

### Penetration of water, dimeticone/cyclomethicone and ethanol into the respiratory system

As far as we are aware, water ingress through spiracles during the immersion process has never been demonstrated. In our study, although all water-immersed head lice showed open spiracles, colored water did not penetrate into the respiratory system (Figure 4i-k). However, the precise mechanism of preventing water penetration has not been elucidated. Nevertheless, we noted the presence of secretions of an unidentified substance in the spiracles of water-immersed head lice (Figure 3c); a similar finding was described by Burgess [10]. Albeit refuted by Ferris [20], Webb [55] described a gland associated with the atrium of lice (atrial glands) that discharges a waxy material in the atrium. Webb suggested the role of dust blockage, but the physical and chemical nature of this substance remains unknown.

In contrast with what was observed following water immersion, the respiratory system of lice immersed in ethanol was flooded within half an hour (Figure 4b). This is in agreement with results reported by Meinking *et al*. with benzyl alcohol [34]; however, the latter suggested that alcohol ingress may have stunned the spiracle in some ways. However, in light of our results, alternative possibilities need to be investigated. Because of its toxicity, flooding of the respiratory system with alcohol results in the death of head lice [37,47,50]. In addition, the dark blue staining observed not only in the respiratory system but in the whole body is due to ethanol ingress through the cuticle of the insect (Figure 4c) as previously described [56].

Dimeticone/cyclomethicone appears to have entered through the spiracles to flood the respiratory system in a similar manner, which results in head lice death within 30 min. Death can be explained by (i) the impossibility for head lice to expel dimeticone/cyclomethicone out of the respiratory system, whereby the insect dies by suffocation as previously reported [48], or (ii) the inhibition of water excretion leading to osmotic stress due to the clogging of spiracles, as described [10]. We used the same formulation (4% dimeticone/cyclomethicone 5, Pouxit^®^) as Burgess [10], and our results appear to confirm the “suffocation hypothesis” proposed by Richling and Böckeler [48] (Figure 4f).

In this study, experiments were performed on head lice collected from several patients from various ethnic communities and the therapeutic history of each patient was not known.

To conclude, a better understanding of the environmental adaptation of lice to the human host may allow us to find improved methods to control head lice.

In this study, we showed that head lice were capable of physiological or behavioral adaptations for surviving anoxia and immersion in water. They can endure anoxia for several hours and are resistant to immersion in water without obvious deleterious effects on movements and survival. Such findings must be taken into account to provide innovative and effective countermeasures against head lice infestation. It appears that efficacy is related to physical entry into the respiratory system that can be easily monitored using dyes.

## Conflict of interest

The authors declare that they have no competing interests.
